# Characterization of a Bioprinted Anticancer Cell Therapy System Generated with Continuous Liquid Interface Production

**DOI:** 10.1002/anbr.202500062

**Published:** 2025-10-02

**Authors:** Lauren Kass, Ike Keku, Yu Zhang, Justin Forbes, Morrent Thang, Jillian Perry, Shawn Hingtgen

**Affiliations:** Division of Pharmacoengineering and Molecular Pharmaceutics UNC Eshelman School of Pharmacy, The University of North Carolina at Chapel Hill 4212 Marsico Hall, 125 Mason Farm Road, Chapel Hill, NC 27599, USA; Division of Pharmacoengineering and Molecular Pharmaceutics UNC Eshelman School of Pharmacy, The University of North Carolina at Chapel Hill 4212 Marsico Hall, 125 Mason Farm Road, Chapel Hill, NC 27599, USA; Division of Pharmacoengineering and Molecular Pharmaceutics UNC Eshelman School of Pharmacy, The University of North Carolina at Chapel Hill 4212 Marsico Hall, 125 Mason Farm Road, Chapel Hill, NC 27599, USA; Division of Pharmacoengineering and Molecular Pharmaceutics UNC Eshelman School of Pharmacy, The University of North Carolina at Chapel Hill 4212 Marsico Hall, 125 Mason Farm Road, Chapel Hill, NC 27599, USA; Division of Pharmacoengineering and Molecular Pharmaceutics UNC Eshelman School of Pharmacy, The University of North Carolina at Chapel Hill 4212 Marsico Hall, 125 Mason Farm Road, Chapel Hill, NC 27599, USA; Division of Pharmacoengineering and Molecular Pharmaceutics UNC Eshelman School of Pharmacy, The University of North Carolina at Chapel Hill 4212 Marsico Hall, 125 Mason Farm Road, Chapel Hill, NC 27599, USA; Center for Nanotechnology in Drug Delivery Eshelman School of Pharmacy, The University of North Carolina at Chapel Hill 2110 Marsico Hall, 125 Mason Farm Road, Chapel Hill, NC 27599, USA; Division of Pharmacoengineering and Molecular Pharmaceutics UNC Eshelman School of Pharmacy, The University of North Carolina at Chapel Hill 4212 Marsico Hall, 125 Mason Farm Road, Chapel Hill, NC 27599, USA; Lineberger Comprehensive Cancer Center, The University of North Carolina at Chapel Hill Chapel Hill, NC 27599, USA

**Keywords:** 3D bioprinting, cell therapies, continuous liquid interface productions, glioblastomas

## Abstract

Anticancer cell therapies have remarkable clinical potential yet fail to reach the clinic due to poor delivery. 3D bioprinting (3DBP) can be leveraged for generating cell therapy delivery devices, where the biomaterial system acts as a protective matrix to stabilize cells after implantation. Continuous liquid interface production (CLIP), an additive manufacturing technology, has several unique features that make it a suitable platform for 3DBP of cell-laden scaffolds. However, the feasibility CLIP bioprinting and efficacy of CLIP-bioprinted cell/matrix therapies have not yet been explored. In this work, we demonstrate the utility of CLIP for cell therapy 3DBP with a simple gelatin methacrylate-based resin and anticancer drug-secreting fibroblasts as a model therapy against recurrent glioblastoma. We demonstrate that CLIP enables rapid, consistent production of cell-laden scaffolds, and cells maintain their viability and tumor-killing efficacy in vitro post-printing. Importantly, we proved that bioprinted cells survive longer in vivo than directly injected cells, and that this effect may correspond to better survival outcomes in a mouse model of glioblastoma resection. This study is the first to utilize CLIP for 3DBP of composite devices containing anticancer cell therapies, providing a crucial foundation for developing highly refined cell therapy delivery devices in the future.

## 1. Introduction

Cell therapies are a therapeutic modality which have been leveraged for treating a variety of pathologies, including cancer.^[1]^ Living cells can respond dynamically to their environment, thus enabling the possibility for a safer and more potent therapeutic effect. While anticancer cell therapies have high potential for clinical success, many ultimately fail in clinical testing due to poor efficacy and safety concerns, which are typically related to inadequate delivery of the cells to the target site.^[2]^ Repeated infusions of systemically delivered cell therapies are required to achieve minimum therapeutic concentrations at the target site, resulting in unstable pharmacokinetic profiles and adverse effects from off-target toxicity.^[3]^ Local delivery of cell therapies can enhance interactions between the cells and their therapeutic target; however, cell therapies may still suffer from poor transplantation efficiency, and rapid clearance from the implantation site is often observed, particularly in postsurgical sites.^[4]^ The development of effective and reliable cell therapy delivery systems is imperative for advancing these highly sophisticated treatment strategies to the clinic.

Biomaterials, such as hydrogel systems, can be used to successfully stabilize cells after local in vivo implantation. These systems act as a protective barrier to cellular clearance, promoting high cell viability and sustaining cellular activity near the site of disease to maximize and prolong therapeutic efficacy. Injectable systems are seen as attractive options for cellular delivery into postsurgical cavities, as these systems conform well to the shape of the surgical defect and can be less invasive than implantation of preformed three-dimensional scaffolds. However, injectable systems are typically prepared immediately prior to clinical use, which means that cells cannot be precultured in the system prior to dosing, despite this “priming” period being vital for in vivo cell survival.^[5]^ Additionally, gelation kinetics must be carefully optimized to prevent premature syringe clogging or insufficient cellular encapsulation upon material deposition in vivo.

In contrast to injectable biomaterial systems, 3D-printed implantable systems have several unique advantages. The shape of the implants can be customized depending on the shape of the surgical defect or to influence the behavior and release of seeded cells. These systems can be easily cultured in vitro prior to implantation, allowing cells to acclimate in their new environment, which greatly enhances their in vivo effect. Finally, these systems can be manufactured and seeded prior to clinical use, ensuring convenience and simplicity at the time of surgery. In a prior study performed by our group, we used 3D-printed biomaterial scaffolds for the delivery of anti-GBM cell therapies.^[6]^ Despite their promising therapeutic effect, these cell-laden scaffolds were seeded with cells attached to the surface of the biomaterial system, which created several challenges in the preparation and implantation of the system. First, a seeding strategy must be developed and optimized for each unique scaffold composition, which is time consuming and may involve extensive postprocessing steps. Second, seeding protocols may involve manual handling of the scaffolds or require seeding be performed one scaffold at a time—both of which can result in significant scaffold-to-scaffold seeding variability. Finally, surface-seeded cells, even after in vitro priming, can be easily disturbed from the biomaterial surface upon exposure to shear forces during in vivo implantation, resulting in suboptimal cellular persistence and efficacy.

Alternatively, cells may be mixed with the 3D printing resin to generate parts containing live cells embedded within the structure. This strategy, known as 3D bioprinting (3DBP), remedies many of the previously mentioned disadvantages associated with external seeding. Cells are incorporated into printed parts in a single step, eliminating the resource- and time-intensive postprint seeding stage. Encapsulated cells can be entrapped in a wider array of materials, as seeding does not rely entirely upon cell-material interactions. 3DBP methods can be broadly categorized as extrusionbased or light-based. In extrusion 3DBP, cells are loaded into a polymer solution which is extruded through a nozzle in a layer-by-layer fashion to build a 3D structure. This method, while relatively simple and inexpensive, is known to be time consuming, and printability is limited.^[7,8]^ Light-based 3DBP systems, which leverage photopolymerization chemistry to crosslink a liquid resin into a 3D shape, are generally more efficient and can generate higher resolution features.^[9]^ However, light-based 3DBP, including methods based on vat polymerization, digital light processing, and stereolithography, incurs several concerns related to cytotoxicity. Cells are exposed directly to unreacted resin components, all of which can cause cytotoxicity at high concentrations, including monomers with reactive functional groups, (i.e., acrylates),^[10-12]^ photoinitiators,^[13,14]^ and, in some cases, light absorbing compounds.^[15,16]^ Systems that utilize light, particularly wavelengths below 405 nm, to catalyze photopolymerization are also associated with UV-induced cellular damage.^[17]^ As a result, it is imperative not only to ensure that cells remain viable postbioprinting but that the cells remain functionally and genetically stable after exposure to the various sources of cytotoxicity.

Continuous liquid interface production (CLIP) is a light-based monolithic 3DP strategy that uses controlled oxygen inhibition of photopolymerization to allow continuous synthesis of a solid 3D part from a liquid photoactive resin.^[18]^ After creating a custom 3D structure using computer-aided design (CAD), the part is created in an inverted fashion with its base layer polymerized onto the upper build platform, which rises as the print proceeds. The window through which UV light is displayed to cure the resin at distinct foci is also permeable to oxygen, which inhibits free radical polymerization in a thin layer above the window known as the “dead zone,” which acts as a continuous source of liquid resin from which the 3D part can be constructed. This promotes more rapid and efficient printing compared to similar 3DP methods, such as stereolithography, in which intermediate processing steps are required due to the layer-by-layer nature of the printing strategy. CLIP has been utilized for various biomedical applications, including our recently published work in which we used CLIP to generate 3D hydrogels externally seeded with anticancer cells for the treatment of glioblastoma (GBM).^[6,19-22]^ To date, the use of CLIP to bioprint structures containing live cells remains unexplored.

In this work, we sought to test the performance of an anti-GBM cell therapy encapsulated in gelatin methacrylate (GelMA) constructs generated via CLIP bioprinting. First, we characterized the seeding efficiency and viability of NHF1 fibroblasts in bioprinted constructs. Next, we tested the stability of the cells postprinting using several functional and molecular characterization assays. Finally, using fibroblasts engineered to constitutively secrete the anticancer protein TNF-related apoptosis inducing ligand (TRAIL) as a model cell therapy, we demonstrated ability for the bioprinted constructs to improve therapeutic outcomes in a model of glioblastoma (GBM) resection in mice compared to controls.

## 2. Experimental Section

### 2.1. Materials and Cell Lines

Gelatin methacryloyl (GelMA, bloom strength = 300, degree of methacrylation = 45–55%) was purchased from Cellink. UV radical initiator lithium phenyl-2,4,6-trimethylbenzoylphosphinate (LAP) was purchased from TCI Chemicals. NHF1 cells were obtained from W. Kauffman (University of North Carolina School of Medicine) and were hTERT immortalized. LN229 and U87 cells were obtained from the American Type Culture Collection. NHF1, LN229, and U87 cells were cultured in Dulbecco’s Modified Eagle Medium (DMEM, Gibco) containing 10% fetal bovine serum and 1% penicillin-streptomycin, hereby referred to as standard culture medium. GBM8 cells were obtained from H. Wakimoto (Massachusetts General Hospital) and cultured in filtered Neurobasal Medium (Gibco) containing 1.5% L-glutamine, 2% B27 supplement, 0.5% N2 supplement, 2 μg mL^−1^ heparin, 20 ng mL^−1^ epidermal growth factor, 20 ng mL^−1^ fibroblast growth factor, and 0.5% anti-anti. All cells were cultured at 37 °C and 5% CO_2_.

### 2.2. Lentiviral Transduction

NHF1 cells were transduced with lentiviruses encoding for green fluorescent protein (GFP) and firefly luciferase (Fluc), hereby referred to as NHF1^Fluc^, or GFP and TRAIL, hereby referred to as NHF1^GFP^ or NHF1^TRAIL^, respectively. LN229, U87, and GBM8 cells were transduced with lentiviruses encoding mCherry (mCh) and Fluc, hereby referred to as LN229^Fluc^, U87^Fluc^, and GBM8^Fluc^. All lentiviruses were purchased from the Duke Viral Vector Core.

Cells were transduced with the lentiviruses at various multiplicities of infection in the presence of polybrene (8 μg mL^−1^) for 24 h in DMEM supplemented with 10% FBS and 1% penicillinstreptomycin. The next day, media was replaced and cells were monitored for fluorescence expression via fluorescence microscopy.

### 2.3. CLIP Bioprinting

CLIP bioprinting was performed using the S1 CLIP prototype printer (Carbon) utilizing a 385 nm LED UV light source. The cylindrical scaffold designs were created in TinkerCAD and exported as STL files which were sliced at 1 μm using the Carbon printing software. Unless otherwise noted, all studies were performed with cylindrical scaffolds of diameter = 3.75 mm and height = 1.5 mm.

The bioprinting resin was prepared by first mixing 10 wt% GelMA, 0.5 wt% LAP, and 89.5 wt% DMEM at 45 °C and 250 RPM until fully dissolved. Next, the GelMA solution was mixed thoroughly in a 1:1 volume ratio with 8 × 10^6^ cells mL^−1^ NHF1 suspension in standard culture medium by pipetting, resulting in final bioresin concentrations of 5% GelMA, 0.25% LAP, and 4 × 10^6^ cells mL^−1^. Scaffolds were printed at a continuous speed of 48 mm h^−1^ and light intensity of 1 mW cm^−2^. Excess resin was removed via gentle washing with deionized water, and scaffolds were immediately placed individually into 12-well plates containing 1 mL fresh standard culture media per well. Scaffolds were cultured at 37 °C and 5% CO_2_ with media changes occurring at 24 h postprinting and every subsequent 48 h.

### 2.4. Quantification of NHF1 Seeding in Bioprinted Scaffolds

NHF1^Fluc^ seeding in bioprinted scaffolds was calculated using a genomic DNA extraction kit (ThermoFisher, K182002). DNA content was extracted per the manufacturer’s instructions from cell suspensions containing 5 × 10^4^–2 × 10^6^ NHF1^Fluc^ to create a standard curve (*n* = 3). DNA concentrations were quantified using the Qubit Fluorometric Quantification system (ThermoFisher). To quantify the number of cells encapsulated per scaffold (*n* = 18 per batch), scaffolds were digested individually with proteinase K at 37 °C for 1 h. Next, DNA was extracted and quantified, and NHF1 cell count per scaffold was determined based on the standard curve.

### 2.5. Viability Assays

#### 2.5.1. Bioluminescence Assay

Bioluminescence was used to compare cell densities in scaffolds printed with different NHF1^Fluc^ concentrations or dimensions. One hour after printing, the scaffolds were placed in a black-walled, clear-bottomed 96-well plate and submerged in 15 mg mL^−1^ D-luciferin (Revvity, 122799) dissolved in 1X phosphate buffered saline (PBS). The samples were incubated in the luciferin solution for 5 min prior to bioluminescence imaging using the in vivo imaging system (IVIS) Spectrum. To measure NHF1^Fluc^ viability in the scaffolds over time, scaffolds cultured for various time points between 0 and 14 days were incubated in luciferin and imaged in the same manner.

#### 2.5.2. PrestoBlue Assay

PrestoBlue reagent (Invitrogen, A13261) was diluted to 1X in 1X PBS. One hundred microliters of the diluted reagent was added to a black-walled, clear-bottomed 96-well plate, and NHF1^TRAIL^-laden scaffolds cultured for various time points between 0 and 14 days were added to the wells (*n* = 3). Wells containing only diluted reagent were used as background controls. The scaffolds were incubated in the reagent for 45 min at 37 °C and 5% CO_2_. Next, the scaffolds were removed from the plate and the fluorescence of the reagent was measured using SpectraMax *M*-series plate reader at an excitation of 560 nm and emission of 590 nm. Results were plotted as absolute fluorescence intensity at each time point with background fluorescence values subtracted.

### 2.6. In Vitro TRAIL Release

Bioprinted scaffolds were prepared as described above with NHF1^TRAIL^ cells and cultured in 12-well plates in 1 mL standard culture media for 1 week at 37 °C and 5% CO_2_. At 1, 3, and 7 days, 500 μL of media was collected and tested using a human TRAIL enzyme-linked immunosorbent assay (ELISA) (Invitrogen, BMS2004) according to the manufacturer’s protocol to determine the concentration of TRAIL secreted into the media. Absorbance was measured using a SpectraMax *M*-series plate reader and compared to a standard curve.

### 2.7. In Vitro Coculture Killing Assays

All coculture assays were conducted using four treatment groups: untreated control, acellular GelMA scaffold, 4 × 10^5^ plated NHF1^TRAIL^, and 4 × 10^5^ NHF1^TRAIL^ in bioprinted scaffolds, prepared as described above. Each treatment was added to a 6-well plate containing 2 mL of standard culture media and incubated at 37 °C and 5% CO_2_ for 72 h. At 72 h, 500 μL of conditioned media from each treatment group was added to a 24-well plate containing tumor cells, which had been plated at a density of 1 × 10^5^ cells well^−1^ (GBM8^Fluc^) or 1 × 10^5^, 5 × 10^4^, and 1 × 10^4^ cells well^−1^ (LN229^Fluc^ and U87^Fluc^) the day prior to treatment. The tumor cells were treated for 24 h before tumor viability was measured via the IVIS spectrum by incubating the cells with 150 μg mL^−1^ luciferin in standard culture media with a 2 min incubation period prior to image acquisition.

### 2.8. Quantification of Reactive Oxygen Species

To quantify reactive oxygen species (ROS) generated during bioprinting, a cellular ROS sensor was used (Abcam, ab186027) according to the manufacturer’s instructions. NHF1^TRAIL^ cells were incubated with 1X PBS containing 0.2% (vol/vol) dye for 30 min at 25 °C on an orbital shaker in the dark. Next, cells were prepared for CLIP bioprinting, UV curing (positive control), or incubation on ice (negative control). Cells were suspended in standard culture media at a concentration of 8 × 10^6^ cells mL^−1^ and placed on ice until processing. CLIP bioprinting was performed as described above. After printing and washing, the scaffolds were placed individually in a black-walled, clear-bottomed 96-well plate containing 100 μL DMEM per well. For UV-cured cells, the cell suspension was diluted 1:1 with 2X concentrated printing resin and then added to a black-walled, clear-bottomed 96-well plate in 100 μL aliquots. The samples were cured in the plate in an LED UV oven (365 nm, 0.6 mW cm^−2^) at 100% intensity for 4 min. Immediately after curing, 100 μL DMEM was added to each well. Untreated cells added in 100 μL aliquots to a black-walled, clear-bottomed 96-well plate containing 200 μL DMEM per well. After treatment, the samples were immediately imaged using a SpectraMax M-series plate reader at an excitation of 520 nm and emission of 605 nm to measure the fluorescence intensity of the ROS dye. A second reading was performed to measure the GFP signal in each well at an excitation of 488 nm and emission of 507 nm. The ROS dye measurement for each well was normalized to the GFP signal in each well to control for the number of cells contained in each sample.

### 2.9. Comet Assay

DNA damage from bioprinting was assessed using a comet assay kit (Abcam, ab238544) according to the manufacturer’s instructions. NHF1^Fluc^ cells were suspended in standard culture media at a concentration of 4 × 10^6^ cells mL^−1^ and placed on ice until processing. Negative control cells were maintained on ice. Cells treated with the CLIP light source were loaded into the printing reservoir and exposed to UV under the same conditions of CLIP bioprinting as described above. Positive control cells were loaded into petri dishes and placed in an LED UV oven (365 nm, 0.6 mW cm^−2^) at 100% (UV high) or 5% (UV low) intensity for 4 min. After treatment, cells were collected and resuspended in cold 1X PBS at a concentration of 1 × 10^6^ cells mL^−1^. Cells were mixed at a 1:10 volume ratio with agarose and pipetted onto a previously prepared comet assay slide containing a thin base layer of gelled agarose. The slides were kept at 4 °C for 20 min to allow the cell-containing layer to form a gel. Next, the slides were immersed in prechilled lysis buffer for 1 h at 4 °C, followed by immersion in prechilled alkaline solution for 30 min at 4 °C. Slides then underwent electrophoresis in alkaline solution for 15 min at 1 V cm^−1^. Slides were washed twice with deionized water, immersed in cold 70% ethanol for 5 min, then air dried. Finally, slides were stained with vista green DNA dye and imaged on an Olympus IX71 fluorescent microscope. Comets were analyzed using the ImageJ OpenComet plugin, and tail moment was calculated for at least 125 cells per treatment group.

### 2.10. Quantitative Reverse Transcription Polymerase Chain Reaction

Quantitative reverse transcription polymerase chain reaction (qRT-PCR) was performed on three treatment groups: untreated control, UV cured samples, and CLIP 3DBP samples. Control cells were plated in cell-culture flasks and cultured at 37 °C and 5% CO_2_. UV-cured samples were prepared as described above with NHF1^Fluc^ cells using a LED UV oven (365 nm, 90 mW cm^−2^) at 5% intensity for 4 min. Cured samples were then cultured in 12-well plates in 1 mL standard culture media at 37 °C and 5% CO_2_. CLIP 3DBP scaffolds were prepared as described above with NHF1^Fluc^ cells and cultured in 12-well plates in 1 mL standard culture media at 37 °C and 5% CO_2_. At days 1 and 7, control cells were harvested from tissue culture plates using trypsin, washed with 1X PBS, and pelleted. For the UV-cured and CLIP groups, 8 scaffolds were pooled per sample per time point. Scaffolds were incubated with trypsin in a 15 mL conical tube on a rotating mixer then centrifuged to release encapsulated cells. Released cells were then washed with 1X PBS and pelleted. Total RNA was extracted from cells using an RNA extraction kit per the manufacturer’s protocol (Invitrogen, 12 183 020) and converted to cDNA (Biorad, 1 708 890). qRT-PCR was performed using the Applied Biosystems QuantStudio 3 Real-Time PCR system with predesigned KiCqStart SYBR Green primers (KSPQ12012) for glyceraldehyde-3-phosphate dehydrogenase (GAPDH), superoxide dismutase 1 (SOD1), heme oxidase 1 (HMOX1), and DNA ligase 4 (LIG4) with PowerUp SYBR Green master mix (Applied Biosystems, A25742). The KiCqStart primer identification numbers are detailed in Table 1. The thermal protocol was 1 cycle at 50 °C for 2 min, 1 cycle at 95 °C for 2 min, and 40 cycles at 95 °C for 15 s followed by 60 °C for 1 min. Relative mRNA expression was quantified using the ΔΔC ΔΔ*C*_*T*_ method using untreated control cells as the biologic control and GAPDH as the endogenous control for each time point.

### 2.11. In Vivo NHF1Fluc Persistence in Bioprinted Scaffolds

Animal studies were approved by the University of North Carolina at Chapel Hill’s Animal Care and Use Committee. Female, athymic nude mice (Animal Studies Core, University of North Carolina at Chapel Hill) aged 6–8 weeks were used for all studies. The animals were first anesthetized using 3% inhaled isoflurane and then placed into a stereotactic frame. The surgical site was prepared using antiseptics betadine and 70% isopropyl alcohol. The skull was exposed with a small incision, and a craniotomy was performed using a microdrill to remove a small portion of the parietal skull plate, 3 mm in diameter, between the bregma and lambda points in the right hemisphere of the brain. Bleeding was controlled using cold saline, and the wound was closed using Vetbond (3M, 1469SB) after bleeding had subsided. Postoperative pain management was performed using subcutaneous injection of 5 mg kg^−1^ meloxicam, 24 and 48 h postsurgery. Five days prior to the implantation surgery, bioprinted scaffolds (*d* = 2.55 mm, *h* = 1.2 mm) were prepared as described above and cultured for 5 days. One week after the craniotomy, implantation of cells or scaffolds was performed. The mice were prepared for surgery as described above, and the surgical site was reopened. The dura mater was removed using a 25 G needle, and a vacuum pump was used to make a mock resection cavity ≈3 mm in diameter and 2 mm in height. Bleeding was controlled with cold saline and GelFoam (Pfizer), when needed. After bleeding had subsided, 1 × 10^5^ NHF1^Fluc^ cells suspended in 3 μL 1X PBS or encapsulated in bioprinted scaffolds (*n* = 4) were implanted into the cavity. The wound was closed with Vetbond and pain was managed via 5 mg kg^−1^ subcutaneous meloxicam 24 and 48 h postsurgery. Serial BLI imaging was performed for 1 month using the IVIS spectrum with 150 mg kg^−1^ intraperitoneal injection of D-luciferin in 1X PBS.

### 2.12. In Vivo Efficacy of NHF1TRAIL in Bioprinted Scaffolds Against GBM8 Resection Model

Craniotomies were performed as described above. One week after the craniotomy, mice were prepared for tumor cell injections. The wound was reopened and 5 × 10^4^ GBM8^Fluc^ cells suspended in 3 μL 1X PBS containing 10% Matrigel were injected into the brain parenchyma using a stereotactic auto-injector. The injections were performed at a rate of 1 μL min^−1^ at stereotactic coordinates 2.5, −0.5, −0.5 from the bregma, avoiding the lateral ventricles. The syringe was slowly removed 2 min after the injection was complete to avoid reflux of the cell suspension. The wound was then closed with Vetbond and postoperative pain was managed via 5 mg kg^−1^ subcutaneous meloxicam injection, 24 and 48 h after surgery. Five days prior to the implantation surgery, bioprinted scaffolds (*d* = 2.55 mm, *h* = 1.2 mm) were prepared as described above and cultured for 5 days. Eleven days after tumor implants, the mice were prepared for tumor resection and stem cell implantation. Prior to surgery, mice were randomized into groups exhibiting statistically similar mean total flux values based on tumor BLI imaging performed immediately preceding resection. The wound was reopened, and tumors were resected using a vacuum pump and fluorescence imaging as guidance. Next, after bleeding had subsided, 1.75 × 10^5^ NHF1^TRAIL^ cells encapsulated in bioprinted scaffolds were implanted into the resection cavity (*n* = 5). Acellular CLIP scaffolds and PBS vehicle injection served as negative controls (*n* = 4). The wounds were closed using Vetbond, and postoperative pain was managed via 5 mg kg^−1^ subcutaneous meloxicam injection, 24 and 48 h after surgery. BLI imaging with the IVIS Spectrum and 150 mg kg^−1^ D-luciferin in 1X PBS via intraperitoneal injection was used to track tumor volume. Mice were euthanized when more than 20% of their original body weight was lost or if other pain-related symptoms, such as dehydration, hunched position, tremors, and low body temperature, were observed.

### 2.13. Statistical Analysis

All results are presented as mean ± standard error of the mean. Batch-to-batch consistency data, in vitro bioluminescence viability data, in vitro coculture killing data, ROS assay data, comet assay data, and qRT-PCR data were analyzed via one-way ANOVA with Tukey’s Multiple Comparisons test. In vivo persistence data was analyzed via Student’s *t* test. Kaplan–Meier survival curves were analyzed using the Log-Rank (Mantel-Cox) test. For all analyses, ns indicates not significant, * indicates *p* < 0.05, ** indicates *p* < 0.01, *** indicates *p* < 0.001, and **** indicates *p* < 0.0001. Statistical analyses were conducted using Prism GraphPad (version 9).

## 3. Results

### 3.1. Fabrication of Bioprinted GelMA Scaffolds with CLIP

**Figure 1.**
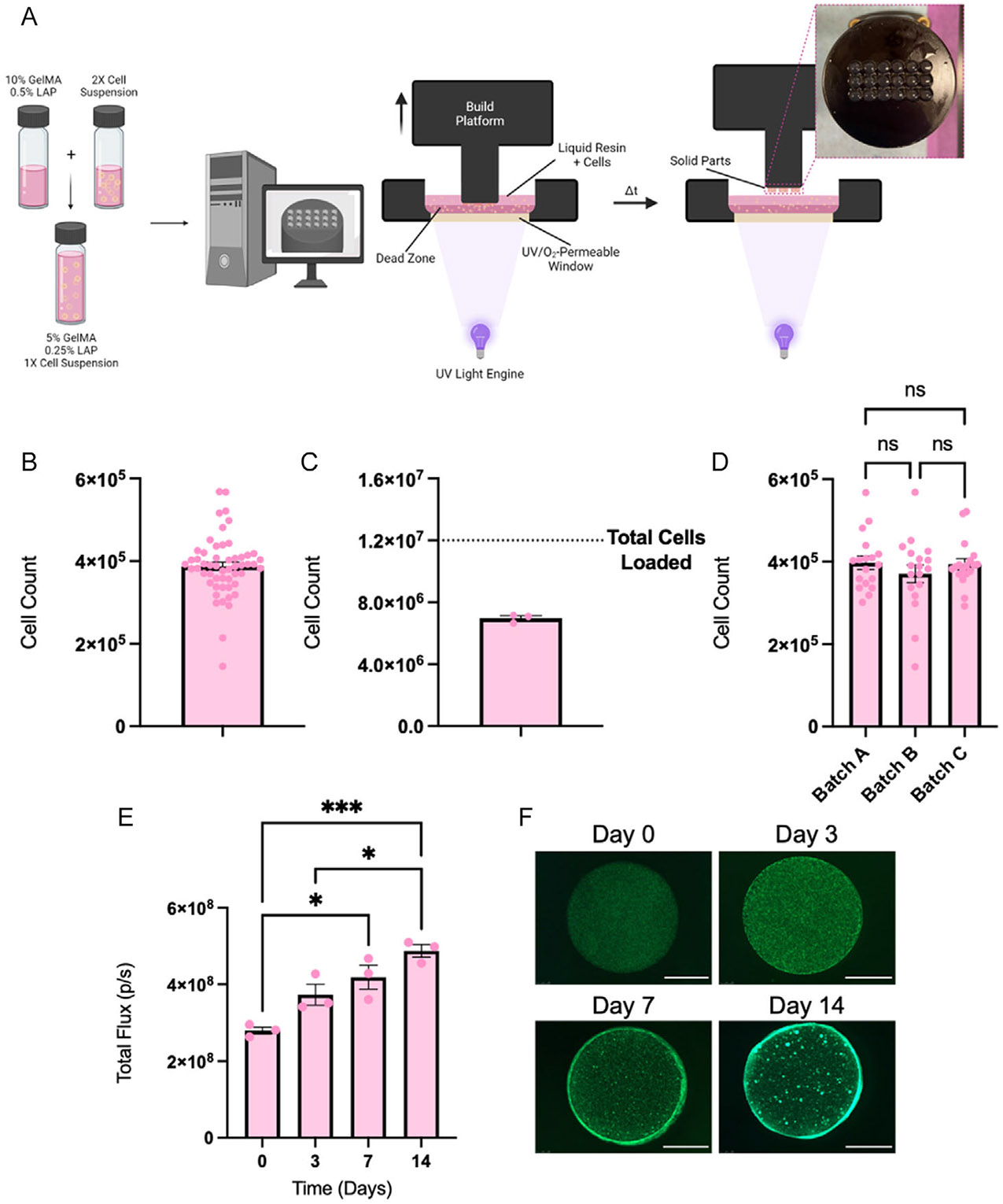
Preliminary bioprinting strategy using CLIP. A) Schematic of CLIP bioprinting strategy. B) NHF1 printed in 3D GelMA constructs at a concentration of 4 × 10^6^ cells mL^−1^, evaluated via DNA isolation (*n* = 18 samples per batch × 3 batches). C) Total of number of NHF1s printed in constructs per batch (*n* = 3, same batches from panel B). D) NHF1s printed in GelMA constructs per batch (*n* = 18 per each batch from panel B). E) BLI signal from NHF1s in bioprinted GelMA constructs at a concentration of 4 × 10^6^ cells mL^−1^ over time (*n* = 3). F) Representative fluorescence images of GFP-positive NHF1s over time in GelMA constructs (brightness and contrast adjusted, scale bar = 1.5 mm).

To explore the 3DBP of live cells using CLIP for the first time, we first sought to develop a simple protocol using a material system with known biocompatibility to determine the feasibility of CLIP-based 3DBP. GelMA is a widely used biopolymer in extrusion and light-based bioprinting systems, known for its biodegradability, cell adhesion properties, and low immunogenicity.^[23]^ We composed a bioresin containing 5% (w/w) GelMA in DMEM with 0.25% (w/w) LAP as a photoinitiator by mixing a solution of 10% GelMA and 0.5% LAP with an equal volume of cell suspension containing 8 × 10^6^ mL^−1^ NHF1^Fluc^ cells in DMEM. This method allowed for rapid and even distribution of the cells in the solution. Immediately following preparation of the cell-laden bioresin, 3 mL of the solution was dispensed into the CLIP printing reservoir, and discshaped scaffolds (*d* = 3.75 mm, *h* = 1.5 mm) were generated (Figure 1A).

We next sought to determine the number of cells encapsulated within individual scaffolds printed in a single batch. To do so, we compared the DNA content of individual scaffolds to a standard curve of DNA concentrations for defined cell counts of NHF1^Fluc^ cells. Cell counts were determined for 3 independently printed batches of scaffolds (*N* = 54, *n* = 18 per batch). An average of 3.87 ± 0.73 × 10^5^ cells were encapsulated in each scaffold (Figure 1B). For each batch of 18 scaffolds, a sum of 6.96 ± 0.73 × 10^6^ cells were encapsulated in scaffolds out of the total 12 × 10^6^ that are loaded into the printer, demonstrating consistency of bioprinting efficiency among independent batches (Figure 1C). Further demonstrating the consistency of the bioprinting process, the data from Figure 1B was separated by batch and compared for statistical differences (Figure 1D). The cell encapsulation per scaffold for batches A (3.94 ± 0.57 × 10^5^ cells), B (3.70 ± 0.92 × 10^5^ cells), and C (3.97 ± 0.67 × 10^5^ cells) were statistically insignificant from one another, indicating that CLIP 3DBP is a strategy that enables the production of consistently seeded scaffolds within and among batches.

After demonstrating the consistency and reproducibility of the CLIP 3DBP process, we next wanted to determine the viability of encapsulated cells over time. Cell viability was quantified with a bioluminescence assay (Figure 1E). The data demonstrates that encapsulated cells proliferate within the scaffold over time, reaching 174% of the initial live cell density after 14 days in vitro. Fluorescence imaging of GFP-positive NHF1^Fluc^ cells confirms the bioluminescence data, with increasing cell densities observed with time in culture. Interestingly, the strong GFP signal around the circumference of the scaffold indicates that cells prefer to grow on the outer edges of the scaffold. Additionally, concentrated foci of bright GFP signal can be observed on days 7 and 14, indicating that cells may be forming clusters as the cell density in the scaffold increases.

### 3.2. Scalability of CLIP Bioprinting

**Figure 2.**
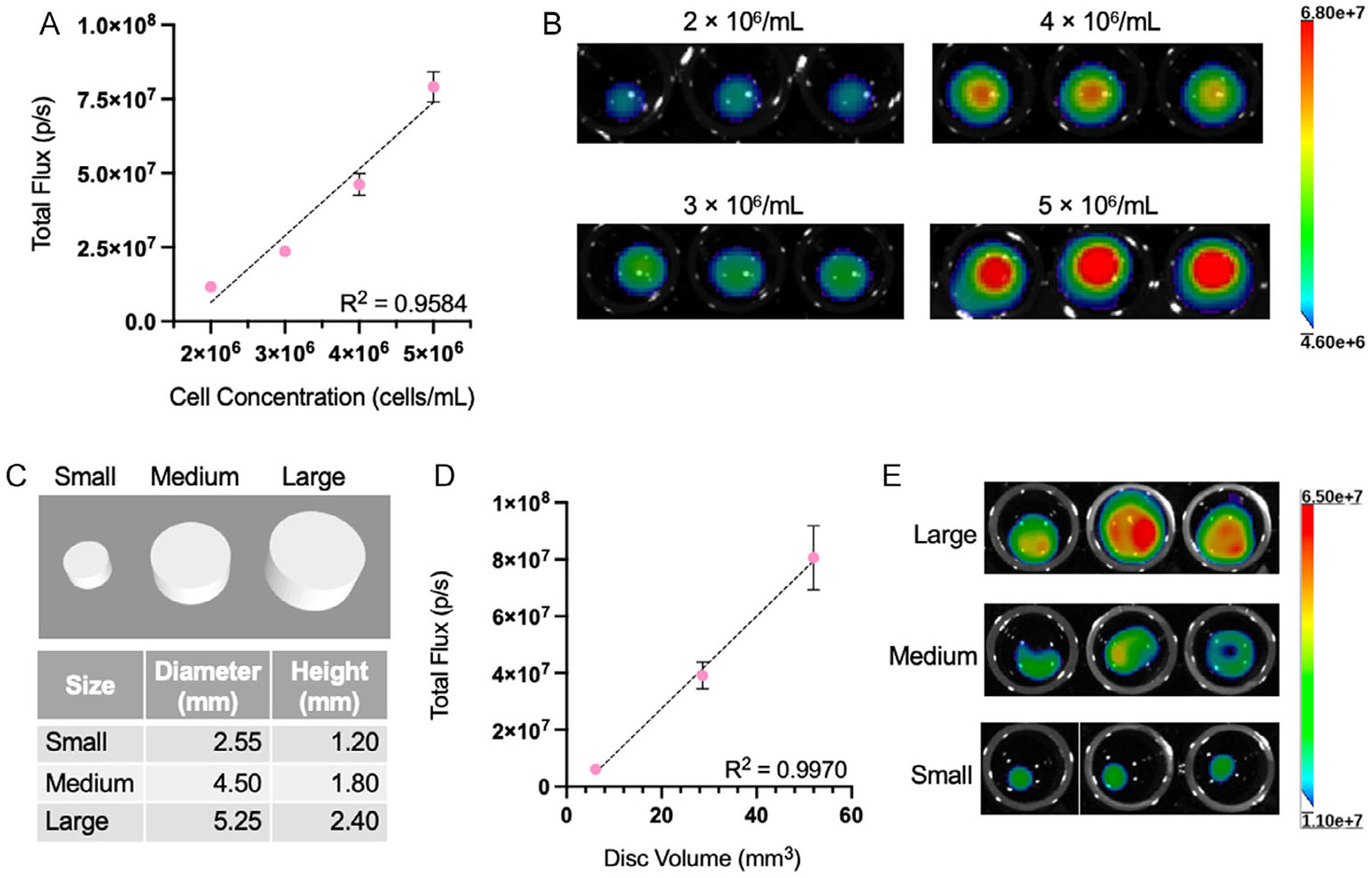
Scalability of CLIP bioprinting. A) BLI signal of NHF1s in GelMA constructs at various concentrations (*n* = 3). B) BLI images corresponding to the data in panel A. C) Images of different sized discs used for CLIP bioprinting. D) BLI signal of NHF1s in GelMA constructs versus volume corresponding to the disc sizes in panel C (*n* = 3). E) BLI images corresponding to the data in panel D.

After confirming that cells can be consistently seeded in bioprinted CLIP scaffolds and that the cells survive for at least 2 weeks postprinting, we wanted to demonstrate the feasibility of adjusting the amount of encapsulated cells in a scaffold by modifying the bioresin cell concentration or scaffold dimensions. We used a bioluminescence assay to quantify the relative density of live cells within each scaffold. We showed that for scaffolds of the same size (*d* = 3.75 mm, *h* = 1.5 mm) printed with different concentrations of NHF1^Fluc^ (2–5 × 10^6^ cells mL^−1^), the bioluminescence signal correlates linearly (*R* = 0.9584) with the bioresin cell concentration (Figure 2A,B). This indicates that initial seeding of cells for scaffolds of the same size and printing conditions can be easily scaled based on the number of cells suspended in the bioresin. Similarly, when scaffolds of different dimensions (Figure 2C) are printed using the same bioresin cell density (4 × 10^6^ NHF1^Fluc^ mL^−1^), the bioluminescence signal increases linearly (*R* = 0.9970) with the volume of the scaffold (Figure 2D,E), indicating that seeding can be easily adjusted by modifying the scaffold dimensions.

### 3.3. NHF1^TRAIL^ Viability and Drug Release from CLIP Scaffolds Bioprinted with Various Bioresin Cell Densities

**Figure 3.**
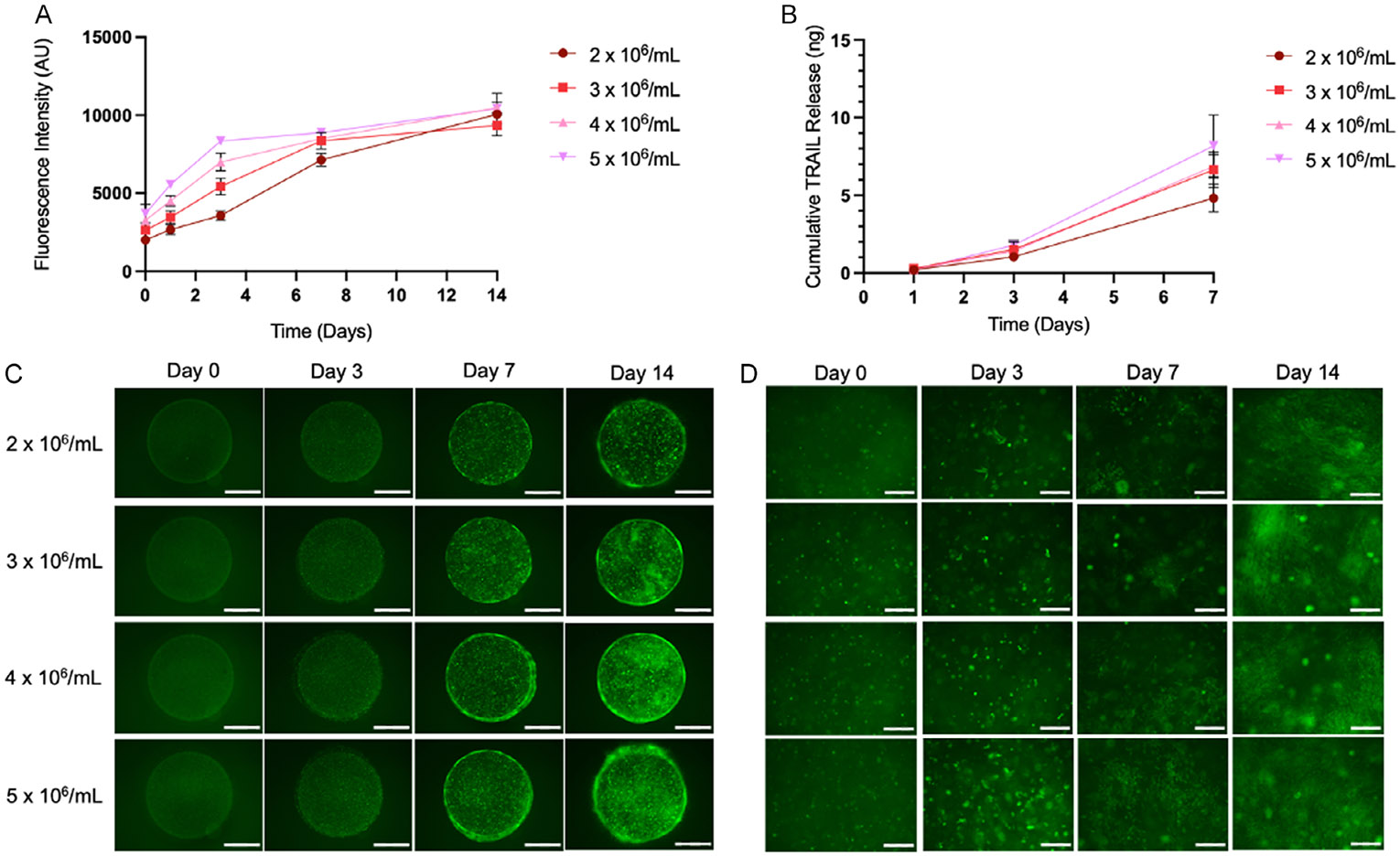
CLIP-bioprinted constructs generated using a range of bioresin NHF1^TRAIL^ concentrations. A) PrestoBlue assay measuring the viability of GelMA-encapsulated NHF1^TRAIL^ cells over time (*n* = 3). B) ELISA-based quantification of secreted TRAIL from bioprinted NHF1^TRAIL^-laden constructs (*n* = 3). C) Representative fluorescence images of GFP-positive NHF1^TRAIL^ cells encapsulated in GelMA constructs at 2X magnification (scale bars = 1.5 mm) and D) 10X magnification (scale bars = 250 μm).

Initial studies were conducted with NHF1^Fluc^ cells. We next wanted to determine the viability of therapeutic TRAIL-secreting NHF1 cells within bioprinted constructs and compare cell growth patterns to cumulative TRAIL release. We performed a PrestoBlue viability assay (Figure 3A) to determine the initial live cell density proliferation rates of CLIP scaffolds printed with different bioresin NHF1^TRAIL^ concentrations. It was found that scaffolds printed with a density of 5 × 10^6^ cells mL^−1^ grew at the fastest rate until day 3. In contrast, the scaffolds printed with a density of 2 × 10^6^ cells mL^−1^ exhibited the slowest growth rate until day 3, at which point the growth rate increased until day 14. All scaffolds reached a similar cell density at day 14; while scaffolds printed with 5 × 10^6^ cells mL^−1^ showed cell growth to 2.8 times its initial density, scaffolds printed with 2 × 10^6^ cells mL^−1^ exhibited growth to 5 times its initial density. The results of the PrestoBlue assay were confirmed with fluorescence imaging. At 2X magnification, the overall density of GFP-positive NHF1^TRAIL^ cells within the scaffold could be visualized (Figure 3C). A bioresin concentration-dependent trend of scaffold cell density was observed on days 0 and 3, whereas at days 7 and 14, the cell densities for different bioresin concentrations appear similar. Again, high GFP signal was observed around the circumference of the scaffold by day 7, indicating that the cells prefer to adhere and proliferate on the exterior edges of the scaffold. Images at 10X magnification demonstrate cell morphology over time in the scaffold (Figure 3D). Observations of cell morphology changes were the same among scaffolds printed with different bioresin concentrations. At day 0, the cells maintain a spherical morphology immediately postprinting. By day 3, cells begin to take on a flattened, fibroblast-like morphology. After day 7, the cells form flat monolayers within the material, appearing similar to the monolayers that form when the cells are grown in 2D tissue culture plastics, demonstrating cell-material interactions with the GelMA scaffold. Spheroid-like clusters of cells can also be observed at this time, suggesting the influence of cell-cell interactions among encapsulated cells.

We next used an ELISA assay to quantify the amount of TRAIL secreted into the media for cultured NHF1^TRAIL^ bioprinted scaffolds over time (Figure 3B). Interestingly, the trend in cumulative TRAIL release does not appear to be dependent on the NHF1^TRAIL^ bioresin concentration. While the average amount of TRAIL released in 7 days is highest for scaffolds printed with the highest bioresin cell concentration (8.18 ng total TRAIL released) and lowest for scaffolds printed with the lowest bioresin cell concentration (4.82 ng total TRAIL released), the difference between these groups is not statistically significant.

### 3.4. In Vitro Killing of GBM Cells with NHF1^TRAIL^-Laden Bioprinted CLIP Scaffolds

**Figure 4.**
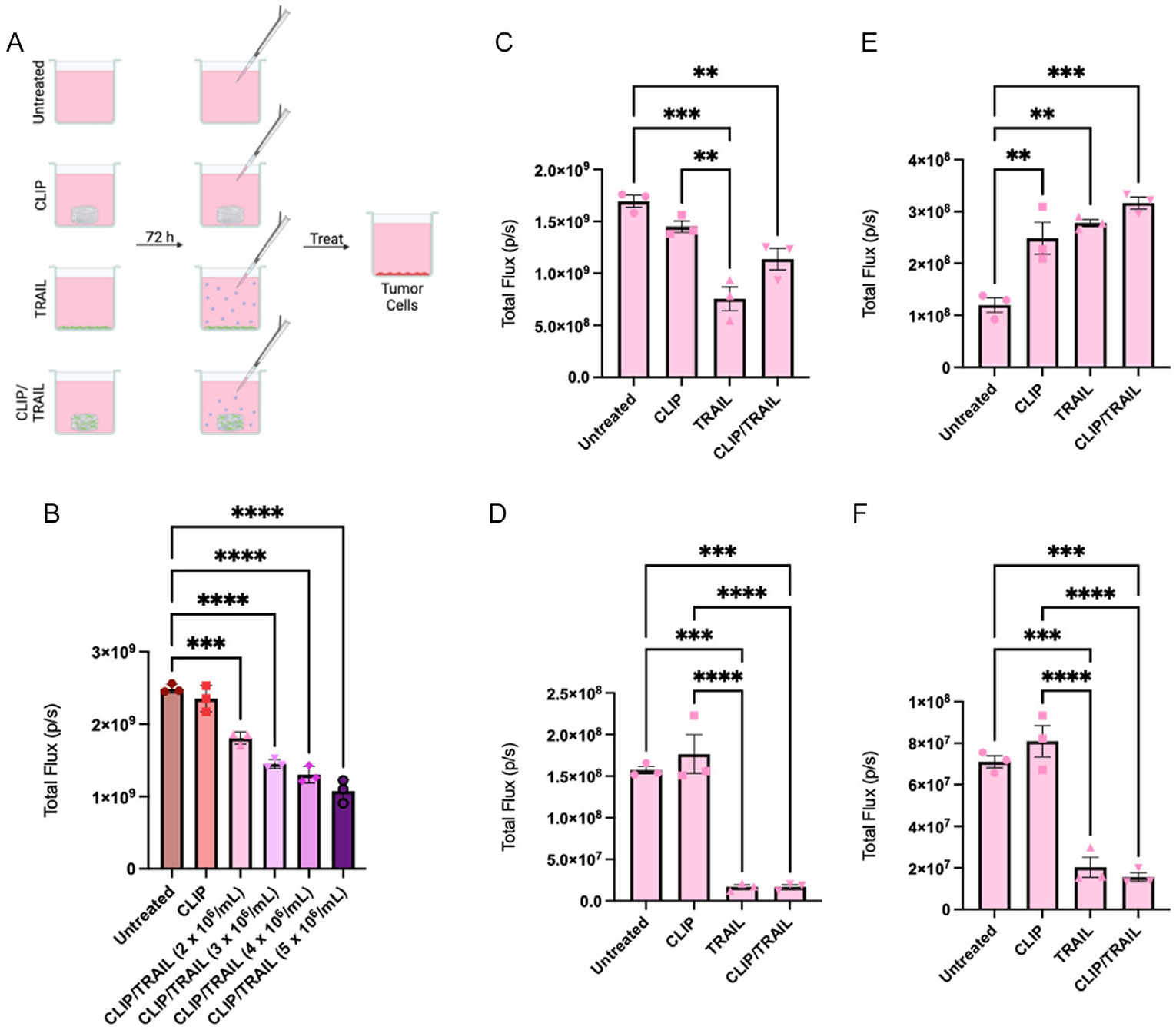
Coculture killing of GBM cells in vitro. A) Schematic of coculture experiment setup and treatment groups. B) BLI signal of GBM8^Fluc^ cells after treatment with TRAIL-conditioned media from GelMA constructs printed with different concentrations of NHF1^TRAIL^ (*n* = 3). C) BLI signal of 1 × 10^5^ LN229^Fluc^ cells after treatment with TRAIL-conditioned media (*n* = 3). D) BLI signal of 1 × 10^4^ LN229^Flu^ cells after treatment with TRAIL-conditioned media (*n* = 3). E) BLI signal of 1 × 10^5^ U87^Fluc^ cells after treatment with TRAIL-conditioned media (*n* = 3). F) BLI signal of 1 × 10^4^ U87^Fluc^ cells after treatment with TRAIL-conditioned media (*n* = 3).

Given confirmation that NHF1^TRAIL^ cells are viable and proliferate after bioprinting, and that secreted TRAIL from bioprinted scaffolds is detectable in culture media, we next sought to determine the therapeutic effect of NHF1^TRAIL^-laden bioprinted scaffolds on GBM^Fluc^ cells in vitro (Figure 4A). We first performed an assay against GBM8^Fluc^ tumor cells, a cell line with known sensitivity to TRAIL (Figure 4B).^[24]^ We showed that culturing of GBM8 cells with TRAIL-conditioned media resulted in increased tumor killing alongside increased NHF1^TRAIL^ concentration in the bioresin. Meanwhile, media conditioned with acellular CLIP scaffolds was nontoxic to GBM8^Fluc^ cells, demonstrating that tumor killing is driven by TRAIL release.

We next wanted to explore how less TRAIL-sensitive tumor lines, LN229^[25]^ and U87,^[26]^ respond to bioprinted NHF1^TRAIL^ therapy, and how this treatment compares to media conditioned by plated NHF1^TRAIL^ cells. We treated LN229^Fluc^ and U87^Fluc^ cells with TRAIL-conditioned media from NHF1^TRAIL^ scaffolds printed at a bioresin concentration of 4 × 10^6^ cells mL^−1^, NHF1^TRAIL^ cells plated at a matched density to the number of cells encapsulated in bioprinted scaffolds, acellular scaffolds, or media only as a negative control. We showed that using conditioned media to treat 1 × 10^5^ tumor cells resulted in significant killing of LN229^Fluc^ by the scaffold-encapsulated NHF1^TRAIL^ cells, though a more robust effect was observed with plated NHF1^TRAIL^ cells (Figure 4C). In contrast, the effect of TRAIL on U87^Fluc^ cells at the same density was antagonistic (Figure 4E). We next performed the same assay against lower tumor cell counts to increase the ratio of TRAIL-secreting cells to tumor cells. At a density of 1 × 10^4^ tumor cells, the difference in tumor killing by scaffold-encapsulated or plated NHF1^TRAIL^ cells was insignificant for both LN229^Fluc^ (Figure 4D) or U87^Fluc^ (Figure 4F) cells, though robust killing by these treatment groups was observed compared to the media-only and acellular CLIP controls.

### 3.5. Molecular Characterization of NHF1 Cells After Bioprinting

**Figure 5.**
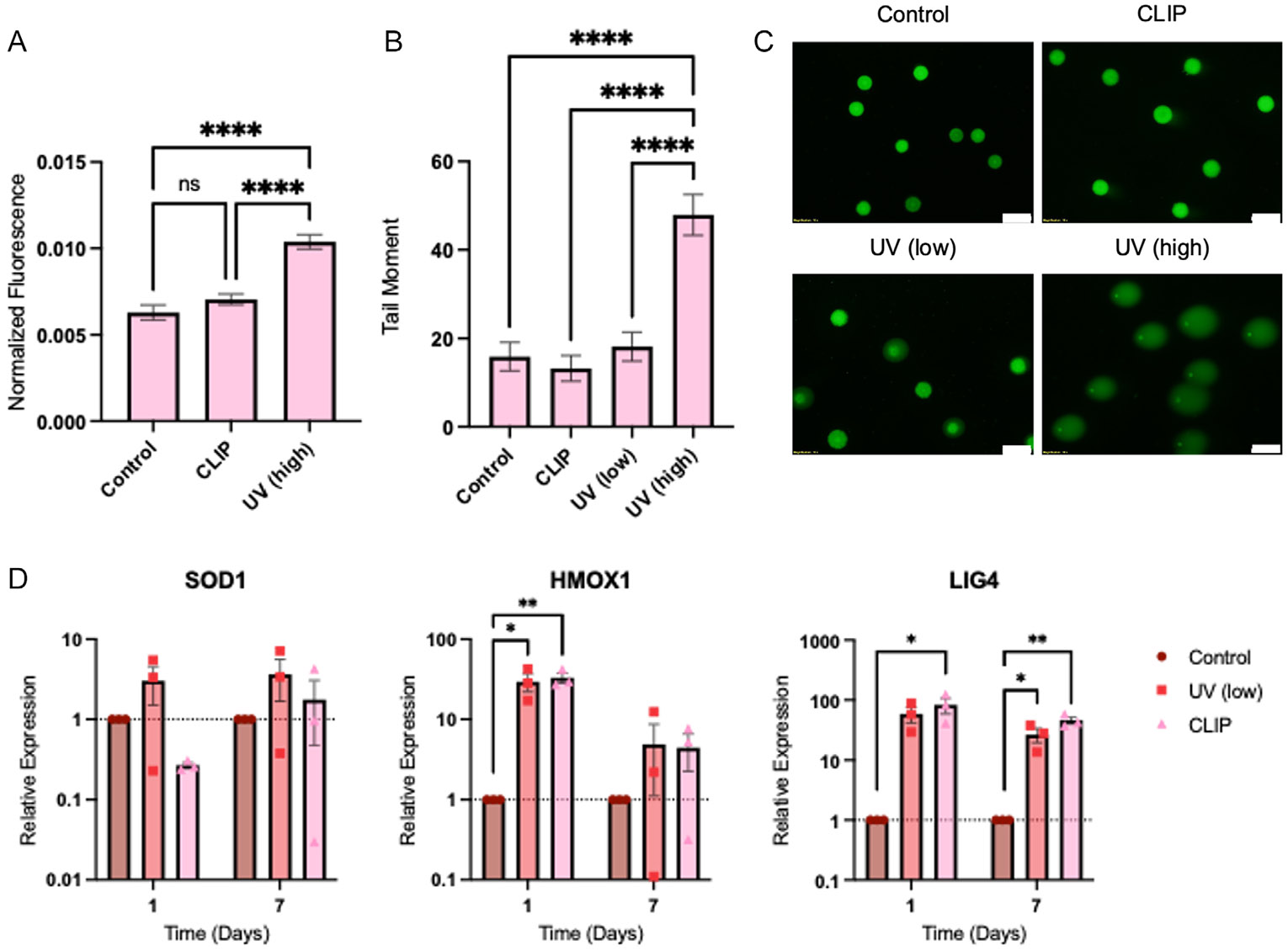
Characterization of NHF1 cell response to bioprinting. A) Quantification of ROS after no treatment (control, *n* = 12), CLIP bioprinting (*n* = 26), and UV curing at high intensity (*n* = 24). B) Quantification of tail moment for each treatment group in the comet assay. C) Representative images of comets in each treatment group from the comet assay (scale bar = 100 μm). D) Quantification of relative mRNA expression levels of SOD1, HMOX1, and LIG4 via qRT-PCR for different treatment groups (*n* = 3).

Though cell viability after CLIP 3DBP is high, we wanted to assess the cellular response to bioprinting on the molecular level using several characterization assays. First, we quantified ROS generated after bioprinting using an ROS sensor dye (Figure 5A). Dye was incubated with cells prior to treatment, after which the dye reacts with ROS inside the cells and emits a fluorescence signal. We found that compared to untreated cells, cells in CLIP bioprinted constructs exhibited a mild but statistically insignificant increase in ROS immediately after bioprinting. Cells cured in the same bioprinting resin using a UV oven with a light wavelength of 365 nm at high intensity was used as a positive control. In these samples, the increase in ROS was statistically significant compared to both the positive control and CLIP 3DBP samples.

Next, a comet assay was performed to assess the DNA damage induced immediately after exposure to various light sources. In this assay, cells were untreated (control) or exposed to the CLIP light source under the same conditions as the previously described bioprinting protocol (CLIP), a sublethal intensity of 365 nm UV light (UV low) for 4 min, or a lethal intensity of 365 nm UV light (UV high) for 4 min. The tail moment, defined as the product of the comet tail length and the comet tail intensity, was reported for at least 125 comets in each treatment group (Figure 5B,C). It was found that the tail moments of the control, CLIP, and UV (low) cells were statistically insignificant to one another. In comparison, the tail moment for the UV (high) cells was increased to 3 times the value of the control cells, a statistically significant result compared to all other treatment groups.

Finally, a qRT-PCR assay was used to assess gene expression of several genes implicated in the cellular response to oxidative stress. SOD1 and HMOX1 are antioxidants, whereas LIG4 is a DNA ligase involved in DNA repair. Cells were harvested from tissue culture plates, from scaffolds generated with CLIP 3DBP as previously described, or from gels generated by UV curing after 1 or 7 days of in vitro culture, and mRNA expression of the three genes was assessed. For SOD1, gene expression for the UV or CLIP treatment groups did not exhibit statistically significant changes compared to the control on day 1 or day 7. However, it was interesting that SOD1 expression in CLIP cells was decreased to 0.27 times the control group expression on day 1. While expression increased to 1.76 times the expression of control cells on day 7, there was more variability in the day 7 samples. In contrast, UV cells exhibited 3 and 3.6 times higher expression of SOD1 than control cells at day 1 and day 7, respectively. For HMOX1, expression was statistically increased for both the UV and CLIP groups (29.4 and 33 times higher, respectively) compared to controls on day 1. On day 7, changes to mRNA expression were statistically insignificant among all treatment groups, though the average expression for the UV and CLIP groups remained higher than controls (4.9 and 4.5 times higher, respectively). LIG4 mRNA expression was significantly increased for the CLIP group and insignificantly increased for the UV group (82.9 and 58.5 times higher, respectively) compared to controls on day 1. Expression was statistically increased in both the UV and CLIP groups (26.6 and 46.2 times higher, respectively) compared to controls on day 7.

### 3.6. Persistence of Bioprinted NHF1^TRAIL^ Cells in Vivo

**Figure 6.**
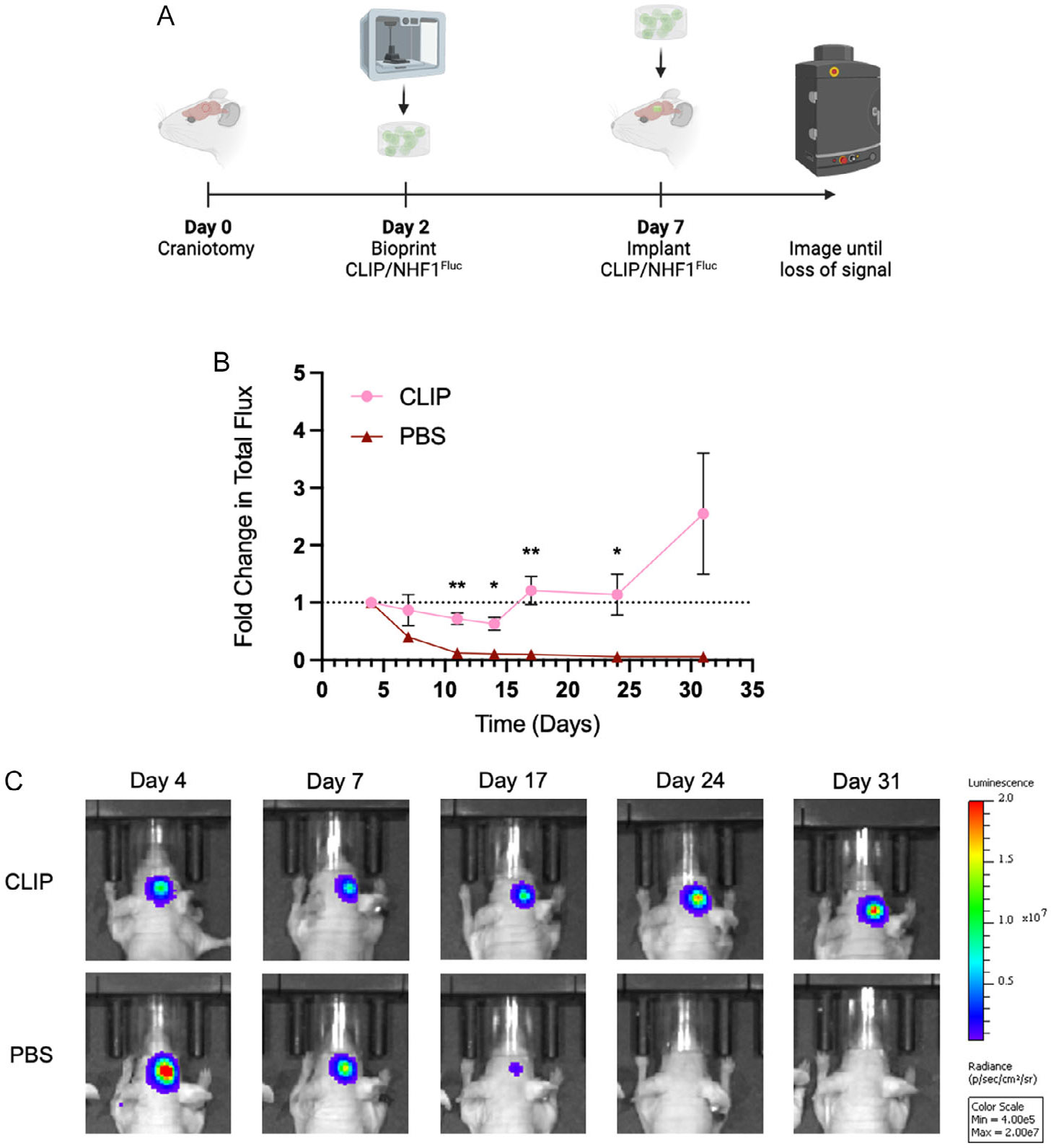
In vivo persistence of NHF1^Fluc^ in bioprinted CLIP constructs. A) Schematic of surgical timeline. B) Fold change in BL1 signals of NHF1^Fluc^ cells over time when delivered in PBS (red, *n* = 4) or bioprinted CLIP constructs (pink, *n* = 4). C) Representative BLI images of mice from each group throughout the study.

The promising in vitro data of NHF1 cells in CLIP-bioprinted scaffolds led us to pursue characterization of the bioprinted scaffolds in vivo. We first wanted to determine if the GelMA scaffold acts as a protective barrier for encapsulated NHF1^Fluc^ cells using a model of mock GBM resection in mice (Figure 6A). Scaffolds with dimensions suitable to fit inside a typical murine brain resection cavity (*d* = 2.55 mm, *h* = 1.2 mm), printed with a bioresin concentration of 4 × 10^6^ NHF1^Fluc^ cells, and precultured for 5 days were implanted into non-tumor bearing mice with resections taken from healthy brain tissue. Mice dosed with 1 × 10^5^ NHF1^Fluc^ cells delivered in saline served as controls. Based on the BLI signal of implanted NHF1^Fluc^ cells, we observed that cells delivered in saline were cleared from the brain rapidly, with the fold change in total flux approaching zero by 11 days (Figure 6B). In contrast, the BLI signal of cells encapsulated in bioprinted scaffolds exhibited a slower rate of decline until day 14, after which the signal began to increase until the study concluded at day 31 (Figure 6C).

### 3.7. Efficacy of Bioprinted NHF1^TRAIL^ Cells in Mice Bearing Partially Resected GBM8 Tumors

**Figure 7.**
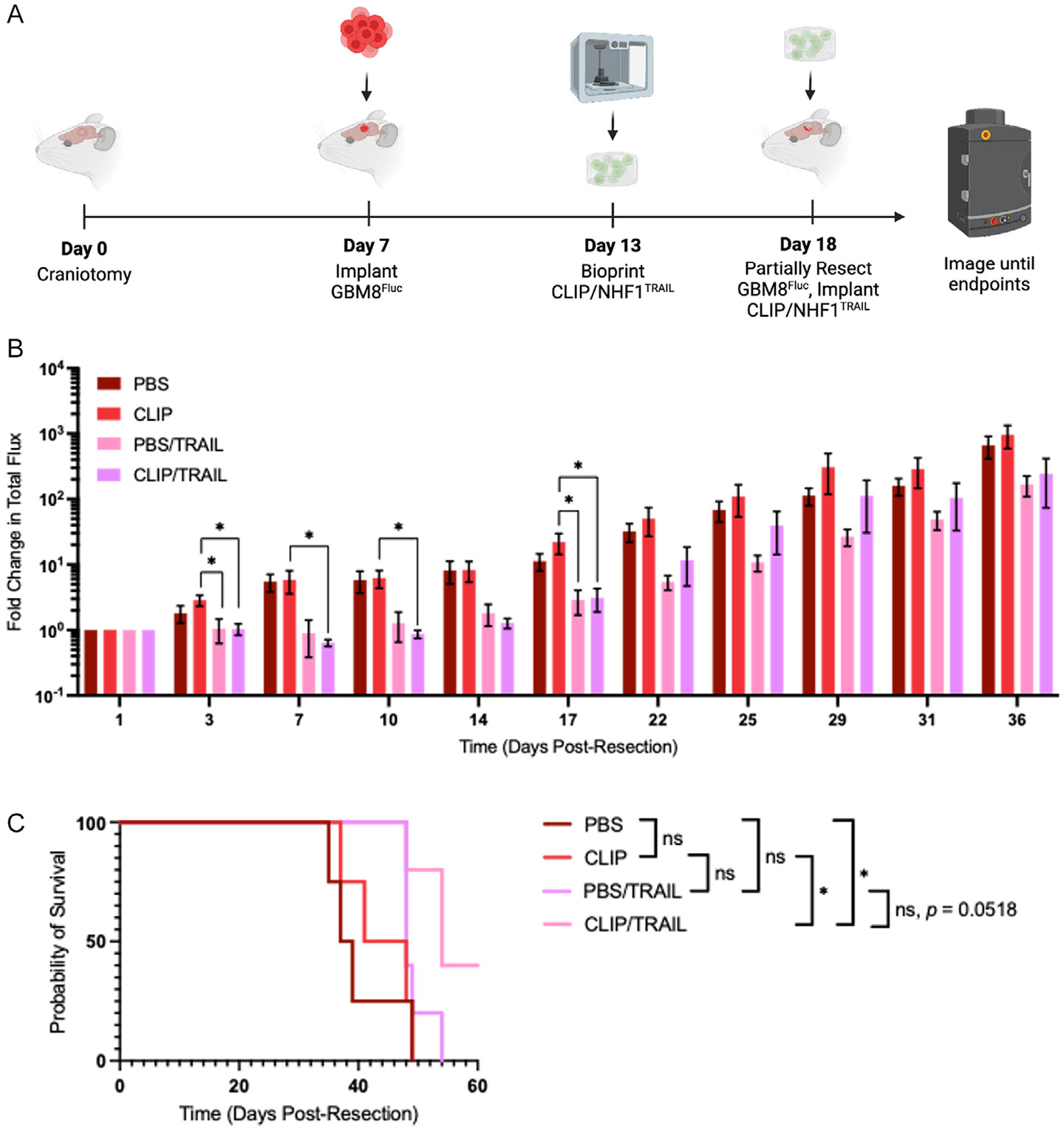
In vivo efficacy of CLIP-bioprinted NHF1^TRAIL^-laden constructs in a mouse model of GBM8 resection. A) Schematic of surgical timeline. B) Fold change in BLI total flux over time for mice treated with PBS (dark red, *n* = 4), acellular CLIP scaffolds (red, *n* = 4), NHF1^TRAIL^ in a PBS suspension (purple, *n* = 5), and bioprinted CLIP scaffolds containing NHF1^TRAIL^ (pink, *n* = 5). C) Kaplan–Meier survival curves for GBM8 tumor-bearing mice.

To determine the impact of NHF1^TRAIL^ encapsulation in CLIP-bioprinted GelMA constructs on tumor-killing efficacy in vivo, we tested the composite scaffolds in mice bearing GBM8 tumors using a model of glioblastoma resection. GBM8 cells were grown in athymic nude mice for 11 days prior to implantation of NHF1^TRAIL^-laden bioprinted CLIP scaffolds, NHF1^TRAIL^ in PBS, acellular CLIP scaffolds, or PBS alone (Figure 7A). Tumor killing was measured via the fold change in bioluminescence signal over time (Figure 7B). While mice in the control groups exhibited immediate and rapid tumor growth postresection, mice treated with bioprinted CLIP/NHF1^TRAIL^ scaffolds exhibited a 2 week delay in tumor recurrence. This delay resulted in a statistically significant improvement in the survival of CLIP/NHF1^TRAIL^ mice (median = 54 days) compared to the PBS (median = 38 days) and CLIP (median = 44.5 days) treatment groups. Though the difference in survival between the PBS/NHF1^TRAIL^ (median = 48 days) and CLIP/NHF1^TRAIL^ group was not statistically significant (*p* = 0.0518), the PBS/NHF1^TRAIL^ group did not significantly improve survival compared to either of the untreated control groups.

## 4. Discussion

Enhancing the delivery of anticancer cell therapies is crucial for easing their translation to the clinic. A myriad of biomaterialbased strategies for encapsulating and delivering therapeutic cells have been published. However, the ideal system would be able to rapidly and reproducibly generate cell-laden structures that can be easily implanted into the patient. Bioprinting is a strategy that could enable the scalable and consistent production of cell therapy delivery devices, as the encapsulation of cells within the polymeric matrix provides protection from external stimuli experienced by the cells in vivo and during manual handling of the device during printing, postprocessing, and surgery. In this work, we sought to develop and validate a strategy using CLIP to demonstrate the effect of cellular encapsulation via bioprinting on the enhanced efficacy of a model cell therapy versus recurrent GBM.

Extrusion 3D printing is the traditional method for bioprinting of cells for tissue engineering or regenerative medicine applications. However, despite the perception that extrusion bioprinting is generally more biocompatible than light-based bioprinting, there is a growing body of evidence in literature to dispute this claim. In fact, many published reports on extrusion bioprinting utilize acrylate-containing photopolymers,^[27,28]^ including GelMA, to generate parts with good shape fidelity—thus exposing cells to toxic functional groups, photoinitiators, and UV light in a similar manner to light-based printing methods.^[29-31]^ Moreover, cells are exposed to shear stress during extrusion, which negatively impacts cell viability.^[32]^ Shear stress increases when higher viscosity resins are used, which are usually needed in order to improve printing resolution.^[33]^ Extrusion printing is also associated with low speed and requires individual parts to be printed one at a time—thus increasing the overall exposure time of cells to toxic monomers or initiators in the resin. In contrast, we demonstrate that CLIP can generate an array of identical, isotropic parts at once in a highly efficient manner, using a resin with low polymer concentration. This permits rapid 3D printing of parts with robust structural integrity and high porosity, wherein toxicity to encapsulated cells is limited and cell growth is maximized postprinting.

In order to demonstrate this application of CLIP, we first identified a resin formulation and printing protocol to encapsulate NHF1 fibroblasts in small discs (Figure 1A). We determined that within and across batches, seeding among the individual discs is consistent—a notable improvement upon individually processed, manually seeded scaffolds (Figure 1B-D). We also demonstrate with a bioluminescence assay that encapsulated cells are viable and can proliferate within the GelMA constructs postprinting (Figure 1E,F), which begins to validate the biocompatibility of the CLIP 3DBP strategy.

A major advantage of 3DBP for cell encapsulation is the ability to readily change the printing protocol or 3D structure to manipulate the number of cells, thus the effective therapeutic dose, within each cell-laden device. We next showed the scalability of CLIP 3DBP when the bioresin concentration or device volume is altered (Figure 2). The linear trends observed in NHF1^Fluc^ bioluminescent signal upon scaling both parameters demonstrate that seeding within the device can be easily tuned. This feature of CLIP is advantageous for producing unique scaffolds among patients that require different implant sizes or cell therapy doses.

We next wanted to thoroughly evaluate the viability of therapeutic drug-secreting fibroblasts within the GelMA constructs over time. We printed GelMA discs with CLIP for a range of bioresin NHF1^TRAIL^ concentrations (2–5 × 10^6^ cells mL^−1^) and evaluated the change in cell viability over time using a PrestoBlue assay. A concentration-dependent effect on the cellular growth rate was observed (Figure 3A), with cells growing slowest in low-density constructs and fastest in high-density constructs until day 3. At day 3, the growth rate cells in high-density constructs slowed, while that of cells in low-density constructs increased, until day 7. By day 14, all constructs had reached a similar cell density, suggesting that the discs had reached the saturation point. Meanwhile, cells exhibited similar morphological features over time regardless of the initial printing concentration (Figure 3C). An interesting trend, maintained across initial printing densities, was that cells preferred to grow around the periphery of the scaffold. A potential explanation for this growth pattern is that cells on the outer surfaces of the material have the most readily available access to nutrients in the media and thus proliferate more quickly in these regions.

TRAIL secretion from GelMA constructs was quantified using an ELISA (Figure 3B). Though the total amount of TRAIL accumulated in media over time followed a printing densitydependent trend, the differences among constructs printed with different initial NHF1^TRAIL^ concentrations were not statistically significant. A potential explanation for this result is that TRAIL is entrapped within the matrix as it is secreted by encapsulated cells, likely by electrostatic interactions between the positively charged TRAIL protein^[34]^ and negatively charged GelMA back-bone.^[35]^ It is likely that the TRAIL accumulation in media is largely owed to the cells growing closer to or on the edges of the scaffold, where effect of protein-biomaterial interactions is minimized. As cells are seen congregating quickly around the outer surfaces of the scaffold regardless of initial printing concentration, the differences in TRAIL secretion are statistically insignificant. This issue could be ameliorated via multiple strategies. First, the resin composition could be optimized to reduce electrostatic interactions with the TRAIL protein, given that the new resin formulation does not negatively impact cell viability or function. Second, the structure of the device could be manipulated to exhibit a higher exposed surface area, which would facilitate increased total release of TRAIL protein into the media. However, for degradable materials such as GelMA, a higher exposed surface area will also theoretically increase the degradation rate of the material, which could result in reduced cellular persistence in vivo.

Despite the possibility of TRAIL secretion being negatively impacted by NHF1^TRAIL^ encapsulation in GelMA, functional coculture tumor killing assays demonstrated that TRAIL concentrations in media conditioned by NHF1^TRAIL^ cells embedded in GelMA constructs via CLIP 3DBP were still therapeutically active. Killing of GBM8 cells was significant compared to untreated controls for NHF1^TRAIL^ cells printed in GelMA at all initial printing concentrations, though a concentration-dependent trend in killing was observed. However, the differences in killing among the NHF1^TRAIL^-containing constructs were statistically insignificant, an expected result considering the previously discussed results of the TRAIL release assay.

Two cell lines with demonstrated resistance to TRAIL, LN229 and U87, were cocultured with CLIP constructs printed at a density of 4 × 10^6^ cells mL^−1^, which translates to ≈4 × 10^5^ total encapsulated cells as demonstrated by the seeding calculations performed in Figure 1. In Figure 4C,E, 1 × 10^5^ LN229 or U87 cells, respectively, were treated with TRAIL-conditioned media from 4 × 10^5^ plated NHF1^TRAIL^, 4 × 10^5^ CLIP-encapsulated NHF1^TRAIL^, or untreated controls. The most robust killing of LN229 cells was observed for the plated NHF1^TRAIL^ group, with CLIP-encapsulated NHF1^TRAIL^ inducing a less potent killing effect due to TRAIL entrapment in the scaffold. In contrast, U87 cells showed an antagonistic response to TRAIL treatment. When the same assay was performed on a lower density (1 × 10^4^) of tumor cells (Figure 4D,F), the killing effect of plated NHF1^TRAIL^ and bioprinted NHF1^TRAIL^ were statistically similar to one another. Collectively, these results indicate that the TRAIL protein secreted from bioprinted NHF1^TRAIL^ is functionally active at therapeutic concentrations for TRAIL-sensitive tumor cells, but the reduction in TRAIL secretion due to protein entrapment by the material may result in insufficient tumor killing, especially if the tumor is TRAIL-resistant.

Cell viability is a common endpoint and criteria for success in studies developing novel bioprinting systems and 3D devices. However, cell viability is not necessarily an indicator of overall cellular health. Bioprinted cells may be alive postprinting, but oxidative stress induced by 3DBP could result in compromised membrane integrity, DNA damage, protein damage, and downstream effects on gene expression, growth rate, or function without causing cell death.^[36-38]^ We sought to investigate some of these molecular responses in cells used in CLIP 3DBP. First, the ROS assay showed that, compared to untreated controls and a UV-cure positive control, cells bioprinted using CLIP exhibited a non-significant increase in ROS (Figure 5A). This result was expected, as cells in CLIP scaffolds will be exposed to UV light and resin components which could induce oxidative stress. However, the results suggest that despite exposure to these sources of toxicity, ROS generation is minimal. Similarly, DNA damage as assessed by the comet assay immediately following bioprinting (Figure 5B,C) was minimal in the CLIP 3DBP group compared to untreated control cells.

Using qRT-PCR, we determined the impact of CLIP bioprinting or UV curing on the mRNA expression of several genes related to the cellular oxidative stress response. HMOX1 expression levels agree with previous reports in literature and expected results of antioxidant expression.^[39]^ On day 1 postbioprinting, UV and CLIP cells exhibit increased expression of HMOX1, indicating that the cells are undergoing an acute oxidative stress response. By day 7, the expression levels are closer to baseline, though still mildly increased. This suggests that the oxidative stress response has dampened after 1 week in culture. However, the results are also more variable at this time point. It would be worth investigating if cells exhibit spatial differences in mRNA expression throughout the 3D structure; for example, cells in the interior of the scaffold, which have limited ability to access nutrients and undergo gas and waste exchange, may undergo a prolonged stress response compared to cells on the exterior of the scaffold. Meanwhile, the other tested antioxidant, SOD1, demonstrates a more complex response. On day 1, UV-treated cells exhibit slightly higher expression of SOD1 compared to controls, while the opposite is true for the CLIP cells. While increased expression of SOD1 in both groups was expected,^[40]^ it has also been reported in literature that SOD1 expression may be dynamic, with early downregulation of the gene, followed by increased expression, then recovery.^[41,42]^ It would be worth investigating this effect on a shorter time scale in future studies to elucidate the differences in SOD1 expression between the CLIP and UV-treated groups. By day 7, SOD1 expression in the UV group is similar to day 1 expression, whereas the CLIP group exhibits an average expression level close to the control group. However, the variability in the CLIP group on day 7 is much higher than it was on day 1, again presenting the possibility for differences in expression across cell populations within the scaffold. Finally, LIG4 expression was increased on both day 1 and day 7 for both the UV and CLIP treatment groups compared to the control. Early increased expression of LIG4 was expected,^[40]^ but the lack of recovery towards baseline was curious. Though both UV and CLIP treatment groups exhibit a slightly reduced expression level on day 7 compared to their day 1 expression, the prolonged increase in expression of LIG4 suggests that DNA repair may be an ongoing process within the bioprinted cells. It would thus be useful to investigate DNA damage and repair kinetics across a broader time scale to determine how much DNA damage is induced post-3DBP and when the DNA repair response returns to baseline—or if the increase in expression is permanent. As errant DNA repair presents the possibility for loss of cellular functions and tumorigenicity, DNA damage and repair should be an important criterion for researchers to assess in the development of biomedical devices containing bioprinted live cells.

We finally wanted to evaluate the in vivo persistence and therapeutic effect of NHF1 cells encapsulated in GelMA via CLIP 3DBP. The in vivo persistence study demonstrated the positive effect of the GelMA scaffold on NHF1^Fluc^ viability over time in the mice (Figure 6B). The reduction in the clearance rate of bioprinted NHF1 cells compared to cells delivered to the cavity directly in PBS was statistically significant between days 11 and 24. This can likely be attributed to the protective effect of the scaffold on the cells against the harsh postsurgical in vivo environment, as well as the positive impact of preimplantation priming period of the cells in the bioprinted constructs. Moreover, we demonstrated that bioprinted NHF1^TRAIL^ cells were able to induce a potent killing response against partially resected GBM8 tumors in mice compared to untreated controls. Mice treated with encapsulated NHF1^TRAIL^ exhibited a period of tumor suppression before recurrence, which corresponded to a significant improvement in overall survival. This result demonstrates that NHF1^TRAIL^ cells encapsulated in GelMA via CLIP 3DBP remain therapeutically active in vivo and suggests that secreted TRAIL from encapsulated cells can diffuse to nearby tumor foci to induce killing. This effect may also be attributed to the positive impact on cellular persistence by the protective scaffold, as NHF1^TRAIL^ cells delivered in PBS were unable to significantly improve survival outcomes compared to untreated controls. However, it is worth exploring the reason that the CLIP/NHF1^TRAIL^ group did not provide significant improvements in mouse survival compared to the PBS/NHF1^TRAIL^ group. This might be explained by the fact that, as shown in Figure 4, TRAIL secretion from unencapsulated cells to nearby tumor cells may be higher than cells encapsulated in hydrogels, due to the diffusion barrier of the material network itself or because of molecular interactions between TRAIL and GelMA, which cause trapping of the protein inside the hydrogel. Even if the cells delivered in PBS are cleared more rapidly than bioprinted cells, it is possible that the concentrations of secreted TRAIL in the resection cavity are higher at early time points, resulting in similar effects in tumor suppression. As such, in the future it will be worth exploring how 3D printing of porous structures could simultaneously improve in vivo cell persistence and cell loading in the cavity while maximizing drug diffusion from the hydrogel for more efficient drug flux to tumor-bearing tissue.

Furthermore, our findings justify exploration into the effect of scaffold degradation kinetics on in vivo persistence and tumor killing. While faster-degrading materials will likely result in a shorter period of cell persistence, cells and drugs will be released more readily to nearby tumor cells; in contrast, slower degrading materials may support a longer duration of cellular persistence, yet further restrict drug release, as these materials usually form networks with smaller inherent pore sizes. Moreover, investigation into the mechanisms of cell clearance from the resection cavity, and how this relates to the kinetics of wound healing in the cavity, could provide valuable insights into the rate at which the scaffold should degrade. Tuning the degradation rate so that cells remain encapsulated until severe inflammation in the cavity has subsided could result in maximum sustained tumor killing.

## 5. Conclusion

The data presented in this work highlight the immense potential for CLIP to be utilized as a biocompatible system for 3DBP of cell therapy delivery devices. Our results demonstrate that anticancer cells encapsulated in GelMA constructs are viable and therapeutically active after CLIP 3DBP. Our molecular characterization begins to explore the cellular response to bioprinting toxicity, though our work presents justification for further studies exploring the ability for cells to recover from oxidative stress post-CLIP 3DBP. Our study also presents opportunities for further development of the CLIP 3DBP strategy, including the development of bioprinting resins capable of generating structures with high-resolution features, the expansion of the platform to bioprint with a variety of therapeutic cell types, and the systematic evaluation of scaffold compositions and architectures to determine how these features influence the behavior of encapsulated cells, drug release from cells in the device, and interactions between the material and host tissue in vivo. Additionally, our work brings forth the justification for refining the CLIP technology specifically for bioprinting—such as building a printer with a light engine in the visible light range or developing a heated reservoir to maintain the cells at physiological temperature—to further improve the biocompatibility of the system. We believe that our work serves as a foundation for the development of clinically relevant cell therapy delivery systems, and though the delivery of an anticancer cell therapy to treat GBM was demonstrated here, this technology could be expanded for use in tissue engineering, synthesis of organoids, development of 3D disease models, the delivery of other anticancer cell therapies, and more.

## Figures and Tables

**Table 1.** qRT-PCR KiCqStart primer identification numbers.

Gene	Species	Gene ID	RefSeq ID	KiCqStart primer pair ID
GAPDH	Human	2597	NM_002046	H_GAPDH_1
SOD1	Human	6647	NM_000454	H_SOD1_1
HMOX1	Human	3162	NM_002133	H_HMOX1_1
LIG4	Human	3891	NM_002312	H_LIG4_1

## Data Availability

The data that support the findings of this study are available from the corresponding author upon reasonable request.
